# A *Fanci* knockout mouse model reveals common and distinct functions for FANCI and FANCD2

**DOI:** 10.1093/nar/gkz514

**Published:** 2019-06-20

**Authors:** Emilie L Dubois, Laure Guitton-Sert, Mariline Béliveau, Kalindi Parmar, Jalila Chagraoui, Julien Vignard, Joris Pauty, Marie-Christine Caron, Yan Coulombe, Rémi Buisson, Karine Jacquet, Clémence Gamblin, Yuandi Gao, Patrick Laprise, Michel Lebel, Guy Sauvageau, Alan D. d’Andrea, Jean-Yves Masson

**Affiliations:** 1 CHU de Québec Research Center, HDQ Pavilion, Oncology Division, 9 McMahon, Québec City, QC G1R 3S3, Canada; 2 Department of Molecular Biology, Medical Biochemistry and Pathology; Laval University Cancer Research Center, Québec City, QC G1V 0A6, Canada; 3 Department of Radiation Oncology, Dana-Farber Cancer Institute, Boston, MA 02215, USA; 4 Laboratory of Molecular Genetics of Hematopoietic Stem Cells, Institute for Research in Immunology and Cancer, Université de Montréal, Montréal, QC, H3C 3J7, Canada; 5 Department of Biological Chemistry, University of California, Irvine, CA 92697, USA; 6 FRQS chair in genome stability

## Abstract

Fanconi Anemia (FA) clinical phenotypes are heterogenous and rely on a mutation in one of the 22 FANC genes (*FANCA-W)* involved in a common interstrand DNA crosslink-repair pathway. A critical step in the activation of FA pathway is the monoubiquitination of FANCD2 and its binding partner FANCI. To better address the clinical phenotype associated with FANCI and the epistatic relationship with FANCD2, we created the first conditional inactivation model for FANCI in mouse. *Fanci* ^−/−^ mice displayed typical FA features such as delayed development *in utero*, microphtalmia, cellular sensitivity to mitomycin C, occasional limb abnormalities and hematological deficiencies. Interestingly, the deletion of *Fanci* leads to a strong meiotic phenotype and severe hypogonadism. FANCI was localized in spermatocytes and spermatids and in the nucleus of oocytes. Both FANCI and FANCD2 proteins co-localized with RPA along meiotic chromosomes, albeit at different levels. Consistent with a role in meiotic recombination, FANCI interacted with RAD51 and stimulated D-loop formation, unlike FANCD2. The double knockout *Fanci^−/−^ Fancd2^−/−^* also showed epistatic relationship for hematological defects while being not epistatic with respect to generating viable mice in crosses of double heterozygotes. Collectively, this study highlights common and distinct functions of FANCI and FANCD2 during mouse development, meiotic recombination and hematopoiesis.

## INTRODUCTION

Generating a mouse model that faithfully recapitulates a human disease represents a fundamental step to better understand disease etiology. This is paramount for rare inherited diseases as they are very difficult to classify and diagnose. Fanconi Anemia (FA) was first described in 1927 by a Swiss pediatrician Guido Fanconi (1). The highly variable clinical manifestations of FA are defined by aplastic anemia, cancer predisposition and developmental defects (i.e. growth retardation, thumb and radial ray defects, hypogonadism, microcephaly, organ dysfunction, infertility and hyperpigmentation) (2,3).

Currently 22 complementation groups have been identified (FANCA, -B (X-linked), -C, -D1, -D2, -E, -F, -G, -I, -J, -L, -M, -N, -O, -P, -Q, -R (dominant), -S, -T, -U, V and -W) (4–7). Together with FA-associated proteins (FAAP24, FAAP100, MHF1, MHF2 and HES1) (5,8–10), FA proteins are involved in a replication-dependent interstrand DNA cross-link (ICLs) repair pathway during S-phase (11), namely, the FA-BRCA pathway. Growing evidence suggests that these FA proteins are also implicated in replication-fork stabilization, stem cell maintenance and cytokinesis. Furthermore, they are involved in combating the deleterious effects of alcohol metabolism (3).

The human protein FANCI (previously known as KIAA1794) has been described as the evolutionarily related partner of FANCD2 (12) as judged by 40% similarity in the region surrounding the conserved monoubiquitinated site (Lys523, corresponding to Lys561 in FANCD2). Upon DNA damage, FANCI and FANCD2 can be phosphorylated by ATM and ATR kinases. The current biochemical model infers that eight FA proteins (FANCA, -B, -C, -E, -F, -G, -L and -M) form the FA core complex with non-FA proteins (FAAP-20, FAAP-24, FAAP-100, MHF1, MHF2 and HES1) (8,13,14). This complex holds a E3 ubiquitin ligase activity via FANCL, which collaborates with the UBE2T ubiquitin-conjugating enzyme (15). After DNA damage, FANCI and FANCD2 proteins are loaded on the altered chromatin (12). Both partners are required for their reciprocal monoubiquitination by the FA core complex (12,16,17). The monoubiquitination of FANCI and FANCD2 is reversed by USP1 (12), leading to the activation of the FA-BRCA pathway by the recruitment of downstream actors such as FAN1, BRCA1 (FANCS), PALB2 (FANCN), BRCA2 (FANCD1), RAD51 (FANCR), RAD51C (FANCO), XRCC2 (FANCU), BACH1/BRIP1 (FANCJ), SLX4 (FANCP) (11,18). These factors lead to the repair of stalled replication forks by exploiting biochemical activities also used in translesion synthesis, nucleotide excision repair (NER) and homologous recombination (HR) (3,13). At a cellular level, monoubiquitinated FANCI and FANCD2 cooperate for the G2/M checkpoint activation and are required for cellular resistance to DNA damaging agents such as mitomycin C (MMC), hydroxyurea, cisplatin and ionizing radiation (IR) (12).

Several FA genes were inactivated individually or in combination in mouse [as reviewed by (4,19–20)]. In 2003, the group of Markus Grompe created a mouse model inactivated in *Fancd2* (21). *Fand2^−/−^* mouse displayed a postnatal growth delay, normal and abnormal testicular tubules and reduced ovarian follicles suggesting meiotic defects. *Fancd2^−/−^* mice also showed exacerbated phenotypes such as microphtalmia, which was present in 78% of animals, and epithelial cancers. Furthermore, a low Mendelian ratio (16.5% instead of the expected 25%) was observed. For a long time, finding the root of the disease, *e.g*. endogenous factors causing genomic instability was a major question in the field. Recent studies suggest that the metabolism of ethanol plays a decisive role in FA (22,23) or in the induction of FANCD1/BRCA2 haploinsufficiency (24). The reactive metabolites derived from ethanol, such as aldehydes, can lead to ICLs when they are not quickly detoxified by aldehyde dehydrogenases (ALDH), which oxidize them in acetate (22). Mice deficient in both aldehyde dehydrogenase 2 and FANCD2 (*Aldh2^−/−^Fancd2^−/−^*) exhibited a more severe FA phenotype, including aplastic anemia, leukemia and congenital defects when compared to *Fancd2^−/−^* mice (22). In addition, alcohol dehydrogenase 5 (*Adh5^−/−^* mice) accumulate formaldehyde adducts in DNA which are repaired in a FANCD2-dependent manner (25).

FANCI and FANCD2 are both involved in the repair of stalled replication forks (16). Further observations indicate two main FANCI-independent functions of FANCD2: as a histone chaperone where FANCI would act only as a modulator (26), and as a regulator of the BLM complex for the recovery of a stalled replication fork (27). Moreover, during replication stress, FANCI and FANCD2 have partially independent roles during the HU- or APH-triggered replication stress response, where FANCD2 mediates cellular resistance to HU or APH independent of FANCI (28). It was reported recently that FANCI functions independently of FANCD2 in ribosome biogenesis (29). In the present study, we created a mouse model to investigate the physiological function of FANCI and to decipher whether FANCI could have independent roles outside of the FANCI and FANCD2 complex (ID2 complex). We show that our *Fanci^−/−^* mouse model recapitulates some phenotypes observed in FA patients, but also highlights common and independent functions to those of FANCD2.

## MATERIALS AND METHODS

### Animal husbandry

All experiments were carried out in accordance to the guidelines of the Canadian Council on Animal Care and approved by the institutional animal care committee (Comité de Protection des Animaux du Centre Hospitalier Universitaire de Québec, CPA-CHUQ; Project/File 2011073-3) and under the approval of the Institutional Animal Care and Use Committee at Dana-Farber Cancer Institute. Both embryonic stem cell clones and animals were genotyped according to the iTL company protocol.

### Mouse strain generation

FANCI conditional mice (*Fanci lox/lox*) were generated in the C57BL/6J strain by the inGenious Targeting Laboratory (iTL). Stably transfected embryonic stem cell clones were selected (by the company) via neomycin resistance gene, flanked by Frt sites and removed by the Flp recombinase. The founder animals received were backcrossed on the pure C57BL/6J strain at least four times. Conditional animals were bred with Sox2 promoter-Cre recombinase transgenic mice (*Cre^+/−^*) on the same background (Jackson Lab stain #008454, *B6.Cg-Tg(Sox2-cre)1Amc/J*). *Fanci^+/−^Cre^+/−^* mice were bred with wild-type mice to remove the Cre transgene and the resulting *Fanci^+/−^* animals were intercrossed to obtain *Fanci* homozygous mutants (*Fanci^−/−^*). The *Fanci^−/−^Fancd2^−/−^* double mutant was generated by crossing the *Fanci*^*+/−*^ mouse with the *Fancd2*^*+/−*^ model of Parmar *et al.* which disrupted Exon 1 (30).

### PCR genotyping

Genomic DNA was extracted from mouse tail samples and used as template for polymerase chain reaction (PCR) genotyping. For PCR reaction, NDEL2 forward primer (5′-GAAACAAACGCGGTACACTTCCTG-3′) and two reverse primers NDEL1 (5′-CCTGTTTCATCCTGCATACTGA-3′) and Lox2 (5′-CTGCCTGTTCTTTGCTTGGTGTGA-3′) generated 500 bp amplicons. The PCR protocol was : 95°C for 2 min and then 20 cycles of 95°C for 30 s, 58°C for 40 s and 72°C for 1 min; last 5 min at 72°C.

A band obtained with NDEL1/NDEL2 primers corresponds to a wild-type allele; Lox2/NDEL2 primers to a mutated allele.

### Northern blot analysis

A premade membrane for Northern Blot from ZYAGEN company (Mouse Multiple tissue Panel NB, Cat MN-MT-2) was used to analyze *Fanci* expression in 22 mouse tissues. Each lane contains 20 mg of total RNA. The confirmation of quality and quantity of ribosomal RNA was provided by the company.

The membrane was first incubated in a hydridization buffer (0.5 M Sodium Phosphate pH7.2, 7% sodium dodecyl sulphate (SDS), 1 mM ethylenediaminetetraacetic acid (EDTA) pH7). Then, P^32^ radioactive-labeled probe covering 900 bp at the 5′ of the mouse *Fanci* gene was used to blot the membrane in the (same) hybridization buffer at 65°C overnight. After blotting, the membrane was washed twice 10 min in saline-sodium citrate (SSC) 2× SDS 0.5%, then twice 15 min in SSC 1× SDS 0.1% and finally twice 10 min in SSC 1× SDS 0.2%.

The 900-bp cDNA probe was generated by a touchdown PCR (from 68 to 55°C for the annealing step, then 30 cycles at 55°C) on wild-type MEFs, using JYM2632 (5′-TTTGGAAGGATCCCGAGCTG-3′) and JYM2663 (5′-AGGCATTTACTGGGATCCCCCTGCTGTCCA-3′) primers.

### Protein extraction from mice tissue

Proteins from bone marrow samples were extracted as previously described (31). Tissue was lysed in 500 μl of ice‐cold lysis buffer (40 mM Tris–HCl pH 7.4, 1% Triton X‐100, 40 mM Beta‐glycero phosphate, 5% Glycerol, 100 mM NaCl, 1 mM EDTA, 50 mM NaF and protease and phosphatase inhibitors (PMSF (1 mM), aprotinin (0.019 TIU/ml), leupeptin (1 μg/ml), NaF (5 mM) and Na_3_VO_4_ (1 mM)) per 100 mg and then crushed using a sterile pestle (Axygen). Lysates were incubated for 30 min on ice and sonicated 30 s ON\OFF for 10 cycles with a Bioruptor (Diagenode). Insoluble material was removed by high‐speed centrifugation at 4°C. Protein extracts were subjected to western blot analysis using anti-FANCI (Bethyl, A301-254A) or anti-FANCD2 (Abcam, ab108928).

### Coronal body MRI imaging

Isoflurane-anaesthetised animals were placed in a mouse bed, their nose facing isoflurane supply (nose cone). Then, the animals were inserted in a dedicated 3.5-cm RF coil, for imaging with a 1-tesla small-animal MRI scanner (ASPECT Imaging, Netanya, Israel). Coronal scans were performed, using a *T_1_*-weighted spin echo 2D sequence, with the following parameters: FOV: 90 mm; 24 slices; 0.9 mm; 0.1 slice gap; dwell time 25 μs; planar resolution 225 μm; fα 90°; TR/TE: 760/18 ms; 1 exc.; 4 min 03 s.

### CT imaging

Tomographic scans were performed *in vivo* on anesthetised mouse using a ExploreLocus 80 Micro-CT (GE Healthcare), with the following parameters : X-ray tube (80 kV), current (450 μA), exposure time (400ms), angular rotation (360°), angular increment (0.5°), voxel (45 μm isotropically). Datasets were reconstructed using reconstruction algorithm provided with the scanner. Analysis was performed with GE Healthcare Microview software.

### Replicative senescence, MMC sensitivity and chromosome spreads

The measurement of population doubling rate, and MMC survival assays were performed as described previously (32).

### Histology and immunostaining

Tissue biopsies were fixed in 4% paraformaldehyde (PFA) over-night, then dehydrated in 70% ethanol and paraffin embedded with successively Leica TP1020 and Leica EG1160 instruments. The 5-μm sections were cut before haematoxylin-eosine or immunohistochemistry stainings (after antigene retrival step). The following primary antibodies were used: rabbit FANCI (SIGMA HPA039972, 1:1000), rabbit FANCD2 (Abcam ab108928, 1:200), WT1 (SantaCruz SC192, 1:400), c-Kit (Dako A4502, 1:600).

### Meiotic spreads

Mouse testes were dissected and preserved in phosphate-buffered saline 1× on ice. Slides were beforehand cleaned by dipping in 100% ethanol with a splash of acetone kept at −20°C. The tunica albuginea was removed from testis and the remaining tubules were kept in 750 μl of sucrose for each wild-type testis. Tubules were then chopped with scalpels. Slides were soaked in fixative solution and kept on the edge of the slide. Then, 10 μl of the testis solution was added on the slide, and it was then tilted to allow the solution to cover the other side of the slide. It was then placed in humid chamber for 45–60 min at room temperature. The cover of the chamber was then removed to allow the solution to dry. Slides were soaked two times in photoflo 0,04%, dried in between. Once the slides were completely dried, they were stored at −80°C and used for immunostaining as described (33). The following antibodies were used: FANCI (rabbit polyclonal, Bethyl A301-254A), FANCD2 (gift from Alan d’Andrea), RPA32 (rat mAb (4E4), Cell signaling).

### Purification of RAD51, FANCI and FANCD2

Human RAD51 was purified as described previously (34). The full length *Fanci* cDNA (generously provided by A. Smogorzewska) was amplified by PCR and cloned in pET52b. The insert, along with 3′-Strep-tag and 5′-His-tag was cloned in pFASTBAC1. Recombinant baculoviruses were produced and used to infect 400 ml of Sf9 insect cells (multiplicity of infection = 10) for 2 days at 27°C. The cell pellet was resuspended in 50 ml of T buffer (50 mM Tris–HCl pH 8.0, 500 mM NaCl, 10% glycerol, 0.02% Triton X-100, 1 mM PMSF, 3 μg/ml leupeptin, aprotinin (0.019 TIU/ml), 5 mM NaF, 1 mM Na_3_VO_4_) containing 5 mM imidazole. The suspension was lysed using a Dounce homogenizer (10 strokes), sonicated four times 30 s and then homogenized a second time. Insoluble material was removed by centrifugation (twice at 35 000 rpm for 1 h in a Sorvall Ultra Pro 80 T647.5 rotor). The supernatant was loaded on a 5 ml Talon column (Clontech) and washed stepwise with T buffer containing 30 mM (75 ml) and 40 mM (25 ml) imidazole. FANCI was then eluted with a 40 ml linear gradient of 0.05–1.0 M imidazole in T buffer. The proteins were identified by SDS-polyacrylamide gel electrophoresis, pooled and dialyzed against R buffer (50 mM Tris–HCl pH 8.0, 10% glycerol, 1 mM dithiothreitol (DTT)) supplemented with 150 mM NaCl (R150) and loaded on a 1 ml HiTrap Q sepharose. The column was washed with 20 ml R150 before a linear gradient of 0.15–1 M NaCl in R buffer was applied. Fractions containing FANCI were dialyzed in storage buffer (20 mM Tris–HCl pH 8, 200 mM NaCl, 10% glycerol, 1 mM DTT, 0.05% Tween 20). When required, the fractions were concentrated using an Amicon ultra-15 column (Millipore) and stored in aliquots at −80°C. FANCD2 was purified as described previously (35).

### Co-immunoprecipitations of purified proteins

Co-immunoprecipitations were performed as (36) using 250 ng of FANCI with 250 ng RAD51 with minor modifications. Recombinant proteins were incubated for 15 min at 37°C in 100 μl of lysis buffer (50 mM Tris–HCl, pH 7.5, 250, 500 or 750 mM NaCl, 0.5% NP‐40) containing protease and phosphatase inhibitors (PMSF (1 mM), aprotinin (0.019 TIU/ml), leupeptin (1 μg/ml), NaF (5 mM) and Na_3_VO_4_ (1 mM)). Proteins were next incubated for 30 min at 4°C with anti-FANCI (Bethyl, A301-254A) in 500 μl of lysis buffer followed by the addition of protein A/G‐sepharose beads (Pierce) for 20 min. Immunoprecipitates were washed four times in lysis buffer and visualized by western blotting using anti-FANCI (Santa Cruz, sc-271316) or anti-RAD51 antibody (Genetex, mAb 14B4).

### D-loop assays

The D-loop assays were conducted essentially as described (37). For substrates with only one 3′-tailed end, the resected DNA was digested with Sca-I restriction enzyme before purification. CsCl-purified pPB4.3 replicative form I DNA (300 μM) was added and the reaction was incubated for 12 min, followed by one-fifth volume of stop buffer (10% SDS and 10 mg/ml proteinase K) and 15 min incubation at 37°C. Labeled DNA products were analyzed by electrophoresis through a 1% TBE1X/agarose gel run at 65V, dried onto DE81 filter paper and visualized by autoradiography.

### Bone marrow hematopoiesis assays

Bone marrow was extracted and analyzed for hematopoietic phenotypes as described (30,38). Briefly, bone marrow was harvested by flushing tibias and femurs from the hind limbs in Hank's balanced salt solution (HBSS) containing 2% fetal bovine serum and 10 mM HEPES buffer (Gibco) (HBSS++). For LSK (Lineage-Sca-1+c-Kit+) staining, cells were stained in HBSS++ using biotinylated-anti-lineage antibody cocktail (BD Biosciences), PE-Cy-7 anti-Sca-1 antibody (BD Biosciences) and APC-anti-c-Kit antibody (BD Biosciences), followed by staining with a secondary PE-Streptavidin (BD Biosciences) antibody. The samples were acquired using a high-speed sorter and data were subsequently analyzed using FlowJo software.

For CFU-C assays, bone marrow cells were seeded in 12-well plates at a density of 50 000 cells/well in methylcellulose medium containing rm stem cell factor, rm IL-3, rm IL-6 and rh Erythropoietin (Methocult GF M3434, Stem Cell Technologies, Vancouver, BC, Canada, http://www.stemcell.com). The cultures were incubated at 37°C in 5% CO_2_ for 7–10 days and macroscopic hematopoietic colonies (CFU-C, colony-forming units in culture) were counted. The triplicate cultures were set up and average was calculated. For CFU-C assays with Mitomycin C, the drug was added in methylcellulose cultures.

### Transplantation assays

Mouse transplantation experiments were performed as reported previously (38). Competitive repopulation experiments using *FancI* knockout mice were performed as follows: 1 million donor (*Fanci^+/+^ or Fanci^−/−^*) Ly5.2 bone marrow cells were transplanted into sublethally irradiated (800 cGy of ^137^Cs gamma irradiation) mice along with 1 million competitor Ly5.1 (*Fanci*^+/+^) cells. Hematopoietic reconstitution was assessed at different time points after transplantation by flow cytometry using anti-ly5-1 and ly5-2-specific antibodies.

## RESULTS

### Generation of a FANCI knockout mouse model

The wild-type murine *Fanci* gene is located on chromosome 7, contains 38 exons covering 58.62 kb with translation starting in exon 2 and shares 80% identity with the human *FANCI* sequence. We generated a conditional mouse knock-out of FANCI and deleted FANCI in the whole organism by crossing the mouse with a Sox2-Cre mouse that expresses Cre in all tissues. The targeting vector, conditional (*Fanci^lox/lox^*) and heterozygous null *Fanci* (*Fanci^+/−^*) mice are described in Supplementary Figure S1A. *Fanci^+/−^* males and females were intercrossed to obtain *Fanci^−/−^* homozygous mice. Offsprings were genotyped by PCR (Supplementary Figure S1B) and Southern blotting to confirm deletion of exons 2 and 3 (Supplementary Figure S1C). Western blotting confirmed loss of FANCI protein (Supplementary Figure S1D) consistent with the loss of exon2 containing the ATG of the *Fanci* gene.

Using a N-terminal probe, the expression pattern of *Fanci* was analyzed by northern blot. *Fanci* was expressed in all tissues, except uterus and lung (Supplementary Figure S2A). The latter was used as a negative control for immunohistochemistry staining. Together with western blots, we were able to confirm the specificity of the FANCI antibody on mouse tissues and that *Fanci* conditional disruption indeed results in null *Fanci* expression (Supplementary Figure S2B).

### *Fanci^−/−^* mice display phenotypes similar to FA

**Figure 1.**
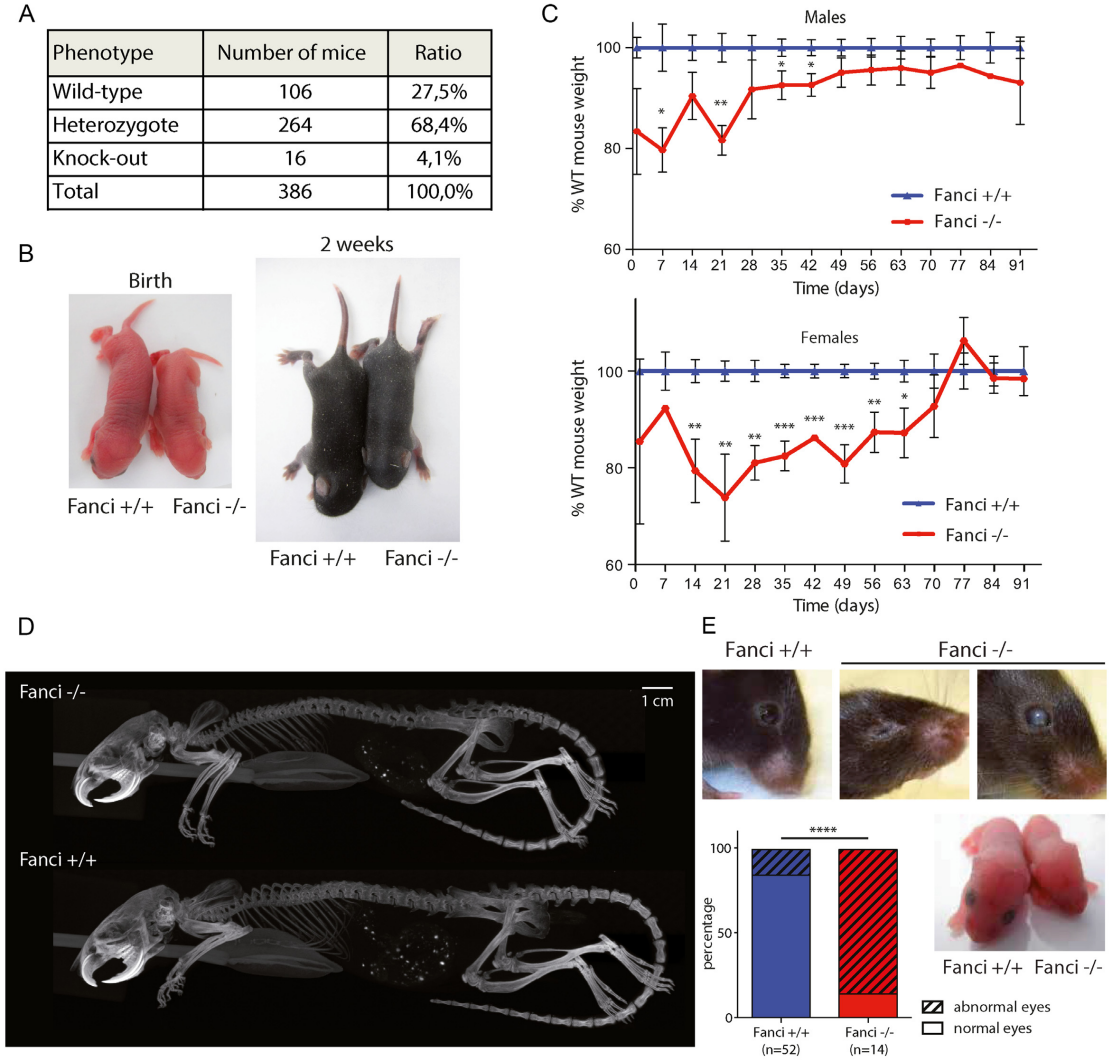
*Fanci^−/−^* animals are smaller and show eye defects. (**A**) Embryonic lethality of *Fanci^−/−^* mice. Genotyping of the litters from heterozygous mating (*n* = 386 mice genotyped). (**B** and **C**) *Fanci^−/−^* mice are smaller. (**B**) Representative picture of *Fanci*^+/+^ and *Fanci^−/−^* new born pups, illustrating the growth delay at birth and 2-weeks-old. Statistical analysis Mann–Whitney (ns: *P*> 0.05, *P< 0.05, **P< 0.01, ***P< 0.001). (**C**) Animal weights of each genotype were followed for 10 weeks. KO males had a 20% growth delay (P < 0.01) compared to the weight of WT males, and females showed a 30% delay (P < 0.005). (**D**) Tomography of two males mice from the same litter at 4 months. The *Fanci^−/−^* mice are smaller but do not show missing vertebraes. (**E**) *Fanci^−/−^* mice have eyes defects. Representative picture of the heads of mice showing anophtalmia, opaque cornea, or impaired development of the lens (Fisher exact test ****P< 0.0001).

Mating heterozygous mice generated only a small number of adult FANCI knockout animals. Genotyping of litters from heterozygous mating (*n* = 386) at weaning age showed a significant sub-Mendelian distribution (27.5% *Fanci^+/+^*, 68,4% *Fanci^+/−^* and only 4.1% *Fanci^−/−^* mice; χ^2^, *P* < 0.0005), suggesting that a proportion of mice died during gestation (Figure 1A). Indeed, similar to *Fancd2^−/−^* mice (21), *Fanci* mutants showed delayed development *in utero*. Reduced size and weight were observed postnatally in *Fanci^−/−^* mice compared to their wild-type siblings (Figure 1B and C). The dwarfism was not due to the absence of any vertebra, as seen on X-Ray analysis of adult mice (Figure 1D). We also observed microphthalmia and impaired development of the lens and retina in *Fanci^−/−^* mice (Figure 1E).

**Figure 2.**
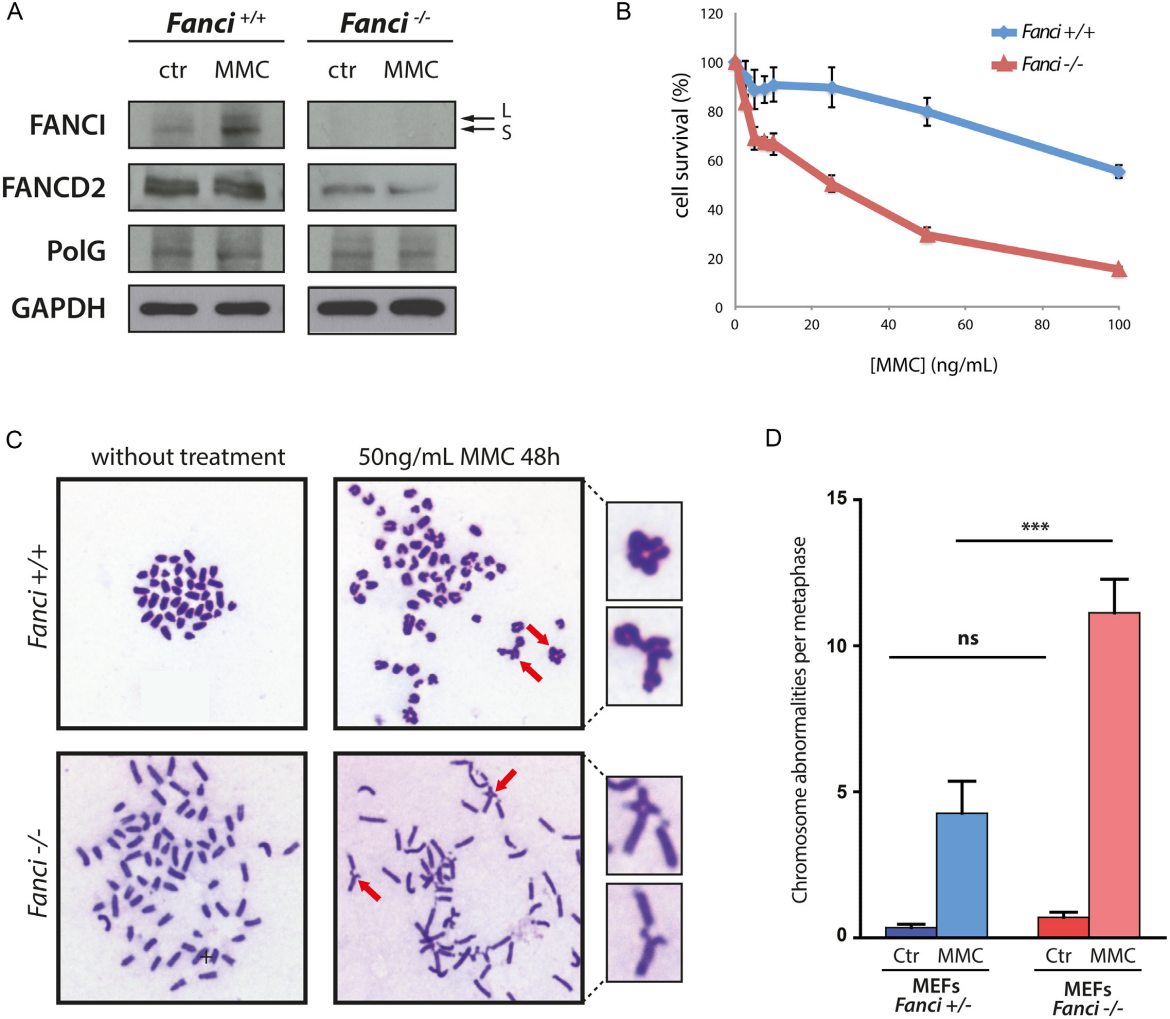
*Fanci^−/−^* MEFs display FA hallmarks. (**A**) Western blot assessing the absence of FANCI in MEFs from *Fanci^−/−^* mice and showing the activation of the FA pathway through FANCI monoubiquitination (upper band (L)), upon 14h treatment with 50 ng/ml of Mitomycin C (MMC). (**B**) Survival assays revealing that *Fanci^−/−^* MEFs, derived from E14.5 embryos, are hypersensitive to MMC crosslinking agent. (**C**) MMC treatment (50 ng/ml for 48 h) induces a high-genomic instability observed in chromosome spread preparations. The red arrows indicate enlarged chromosomal abnormalities. (**D**) Quantification of chromosomal aberrations. Mann–Whitney statistical analysis was performed (ns *P*> 0.05, ***P< 0.0001).

A characteristic phenotype of the FA pathway mutants is the hypersensitivity of FA cells to DNA crosslinking agents. We derived primary mouse embryonic fibroblasts (MEFs) from E14.5 embryos, and monitored cell population doublings as well as sensitivity to MMC. As expected, FANCI was monoubiquitinated after drug treatment in wild-type cells (Figure 2A). Ubiquitinated FANCD2 was absent in *Fanci^−/−^* MEFs (Figure 2A) and FANCD2 protein levels were severely decreased in *Fanci* knockout mice. This is in line with the observation that FANCD2 and FANCI are partially interdependent for their protein stability (28). *Fanci^−/−^* MEFs displayed growth retardation and replicative senescence (Supplementary Figure S3), and were hypersensitive to MMC (Figure 2B), compared to wild-type MEFs. Spontaneous chromosome breakage and quadriradial chromosome formation were observed (Figure 2C), and the average number of abnormal chromosomes was even higher following MMC treatment (Figure 2D). These observations are in accordance with the signature of FA cells (39).

### Hematological status in *Fanci*^−/-^mice

**Figure 3.**
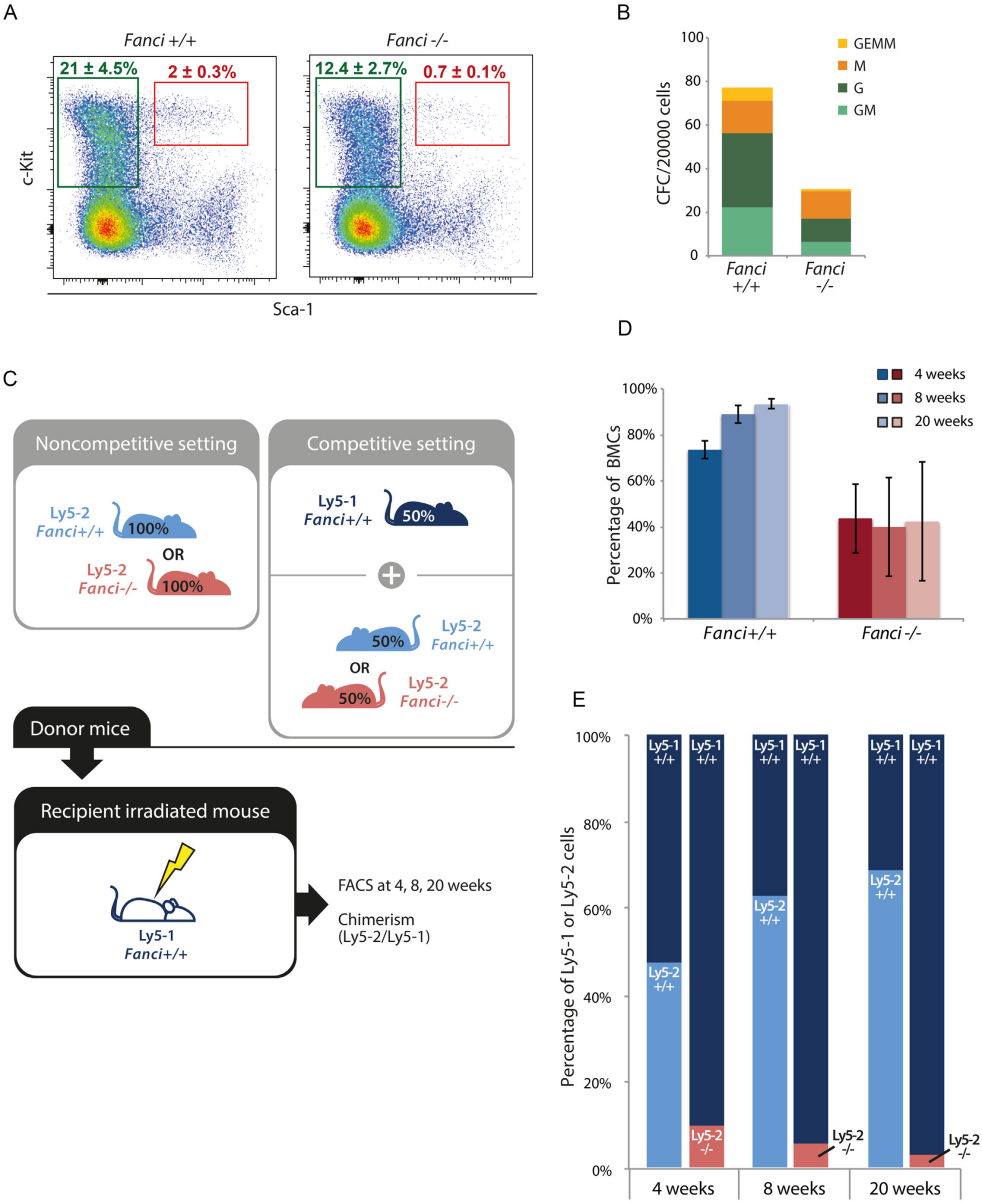
*Fanci^−/−^* mice show bone marrow hematopoietic defects. (**A**) Bone marrow cells from *Fanci*^+/+^ and *Fanci^−/−^* animals (*n* = 4 per genotype) were analyzed by FACS. Green gates contain Lin- Sca1- c-Kit+ (LK) cells, corresponding to the progenitor-enriched population. Red gates surround Lin- Sca1+ c-Kit+ (LSK), known as the HSC-enriched population. Each plot represents a total of 2 × 10^6^ events. (**B**) Bone marrow cells from the same animals were plated in a methylcellulose medium for a CFC. The numbers of Colony-Forming Unit-Granulocyte, Erythroid, Monocyte and Megakaryocyte progenitors (CFU- GEMM), CFU-GM (granulocyte and monocyte progenitors), CFU-M (monocyte progenitors) and CFU-G (granulocyte progenitors) were counted after 7 days of incubation, from 20 000 *Fanci^−/−^* or syngenic wild-type bone marrow cells plated in triplicate. (**C**) Transplantation strategies with and without competition. (**D**) Bone Marrow Cells (BMCs) were harvested from *Fanci*^+/+^ or *Fanci^−/−^* littermates. These Ly5-2 BMCs were transplanted in Ly5-1 wild-type recipient mice. (**E**) Transplantation in competition. After 4, 8 or 20 weeks post-transplantation, the proportion of Ly5-2 cells was evaluated in the peripheral blood of the recipients.

As systematically described for deficiency of FANC proteins (39,40), we analyzed whether FANCI is involved in hematopoietic stem cell (HSC) function. First, we assessed the cellularity of the HSC compartment. Tibiae, femurs and pelvis were isolated from 6- to 9-month-old *Fanci^+/+^* and *Fanci^−/−^* male littermates. Bone marrow cells were stained for HSC (Lin-, c-Kit+, Sca1+, CD48-, CD150+) and analyzed by flow cytometry. Lin-CD48+CD140-cells were reduced by 22% in *Fanci*^−/−^ mice compared to wild-type littermates (0.33 ± 0.08 versus 0.42 ± 0.12, respectively) (Supplementary Figure S4A). LK cells (Lin- c-Kit+, strongly enriched for progenitor cells) were reduced by 41% of wild-type levels in the *Fanci^−/−^* marrow (*n* = 4 per genotype). Strikingly, the phenotype of Lin- Sca1+ c-Kit+ cells (LSK, Figure 3A) appeared even stronger with a 65% reduction. Mono-potent to multi-potent myeloid colony forming cells (CFCs) were accordingly reduced in *Fanci^−/−^* mice (Figure 3B). Noticeably the proliferative potential of these CFC was markedly reduced, as much smaller colonies were obtained from the mutant cells when compared to wild-type (Supplementary Figure S4B).

To further investigate the hematopoietic defect of *Fanci^−/−^* mice, *in vivo* functional studies were performed (Figure 3C). In a first series of experiments, cells were transplanted in noncompetitive set up. CD45.2 bone marrow cells from 6-month and older *Fanci^+/+^* or *Fanci^−/−^* littermates were transplanted in congenic (CD45.1) irradiated recipients. At different time points (4, 8 and 20 weeks), we appraised the engraftment level of these CD45.2 cells by ascertaining the peripheral blood chimerism. At every time points analyzed (4–20 weeks after transplantation), *Fanci^−/−^* cells reconstituted at best 50% of the blood of recipient mice whereas control grafts injected at similar cell dose almost reached 95% reconstitution by 20 weeks (Figure 3D and Supplementary Figure S4C). To further assess the reconstitution potential of *Fanci^−/−^* bone marrow, competitive transplantation experiments were designed in which case half the graft is CD45.1 (competitor and wild-type for *Fanci*) and the other half CD45.2 homogygous null mutant or wild-type. By providing control CD45.1 graft in all recipients, this set up provides a more detailed assessment of the *Fanci^−/−^* grafts in less stressful conditions as the control CD45.1 cells secures short and long-term hematopoiesis. In these experiments, *Fanci^−/−^* cells, although representing 50% of the graft, could never produce more than 10% of the nucleated blood cells in recipient mice (right bars in Figure 3E and Supplementary Figure S4C). In sharp contrast, reconstitution reached up to 70% levels in competitive experiments performed using the same mixture of CD45.1 competitor + CD45.2 wild-type cells (left bars in Figure 3E and Supplementary Figure S4C).

Collectively, these results suggested that *Fanci^−/−^* HSCs have a limited ability to reconstitute the hematopoietic compartment and cannot adequately compete with wild-type cells. Despite this limited capacity of repopulation, *Fanci^−/−^* HSCs seem, however, able to form a bone marrow by generating progenitors and all cell lineages, thus allowing a fraction of *Fanci^−/−^* mutant mice to survive.

### Interbreeding FANCD2 and FANCI deficient mouse models and epistatic relationships for hematopoietic defects

**Figure 4.**
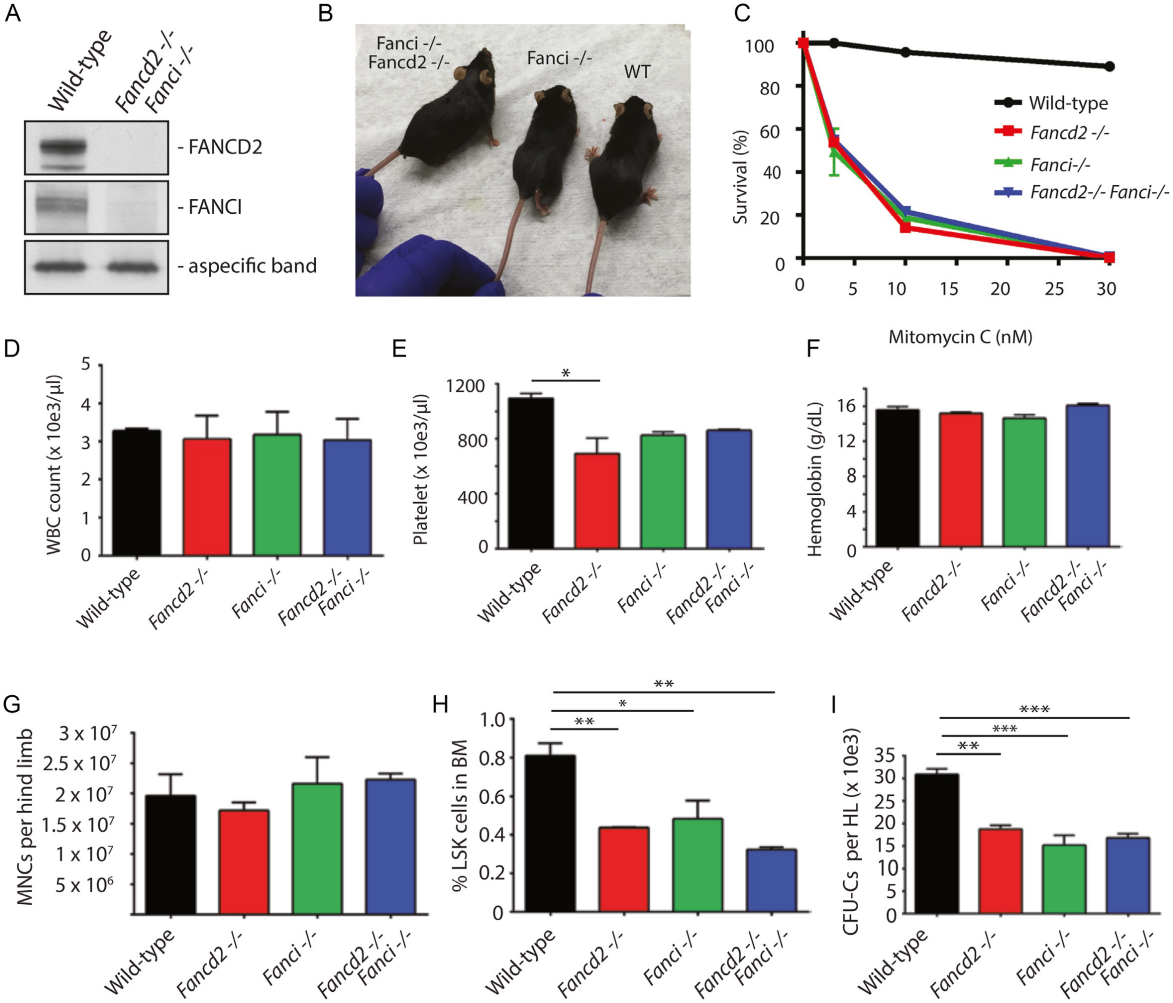
Combined inactivation of both FANCI and FANCD2 is epistatic for bone marrow hematopoetic phenotypes. (**A**) Western blot analysis of FANCD2 and FANCI proteins in *Fanci^−/−^Fancd2^−/−^* knockout mice. (**B**) *Fanci^−/−^Fancd2^−/−^* mice are viable. The mice shown are 4 to 5 months old females. (**C**) Bone marrow progenitors from single KO mice or double KO mice are hypersensitive to Mitomycin C. Bone marrow cells were cultured in triplicates in methylcellulose medium containing Mitomycin C for 10 days in CFU-C assay and the number of hematopoietic colonies were counted. (**D-I**) Wild-type, *Fancd2^−/−^, Fanci^−/−^* or the double knockout *Fancd2^−/−^Fanci*^−/−^mice were analyzed for (**D**) white blood cell (WBC) count, (**E**) blood platelet count, (**F**) blood hemoglobin levels, (**G**) bone marrow cellularity, (**H**) percentage of LSK cells in the bone marrow, and (**I**) bone marrow colony-forming unit cells. The data in panels D–I are from *n* = 3–4 mice per group except in panels D-F in which the group size was *n* = 2 for *Fancd2^−/−^Fanci^−/−^* mice. *P*-values were determined by oneway Anova analysis (multiple comparisons) using a Prizm graphpad. Only statistically significant comparisons are shown (*P≤ 0.05, **P≤ 0.01, ***P≤ 0.001).

In order to assess the functional relationship between FANCI and FANCD2 proteins in bone marrow hematopoiesis, we generated double-mutant *Fanci^−/−^Fancd2^−/−^* mice in the C57BL/6J background. Western blot analysis showed an absence of FANCI and FANCD2 proteins in *Fanci^−/−^Fancd2^−/−^* mice (Figure 4A and Supplementary Figure S5A). The double mutant *Fanci^−/−^Fancd2^−/−^* mice were viable but were born at much lower Mendelian frequencies than expected (Supplementary Table S1), and with no growth delay at 5 months (Figure 4B). As expected, bone marrow hematopoietic progenitors from all mutant mice were hypersensitive to mitomycin C, with no difference between the cells from single versus double knockouts (Figure 4C).

**Figure 5.**
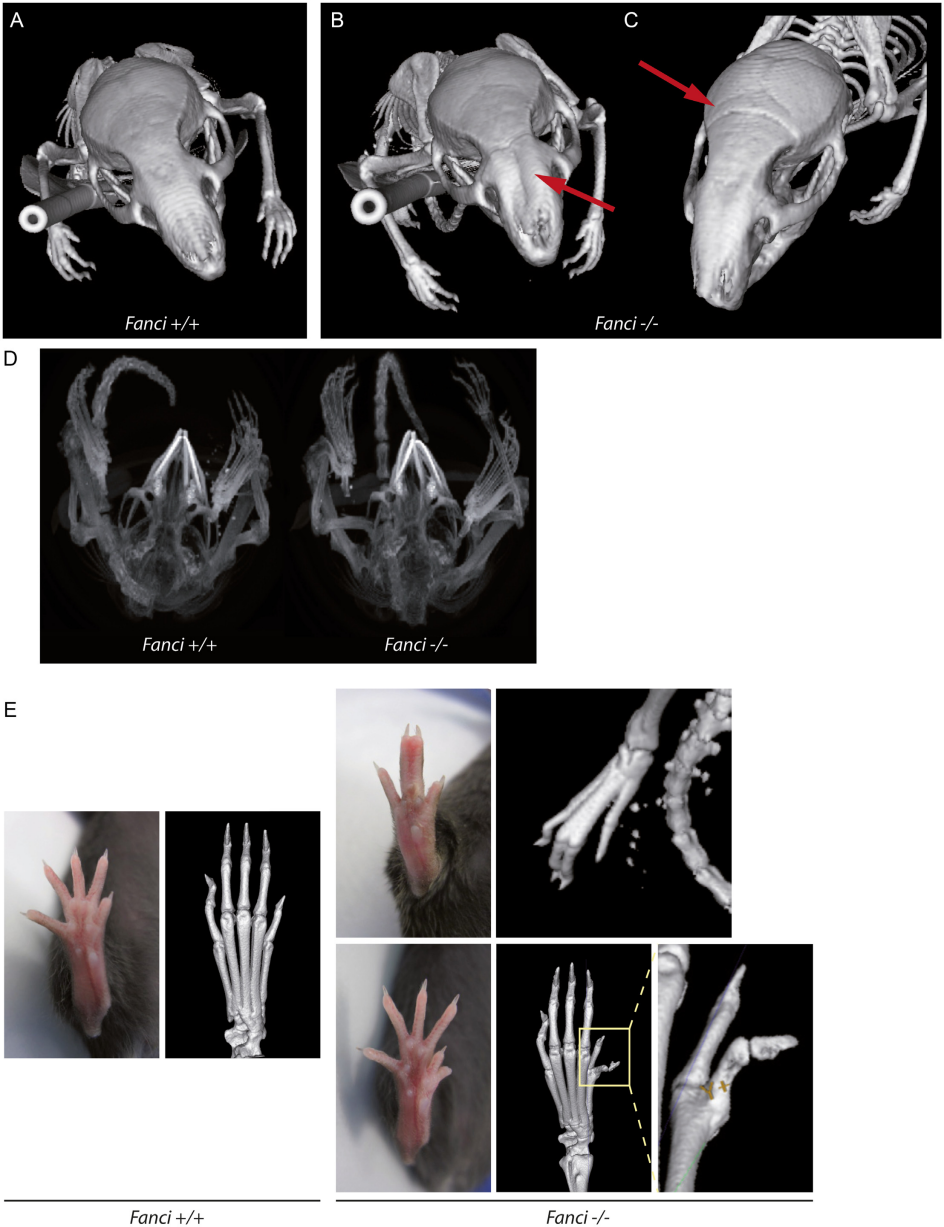
*Fanci^−/−^* mice show skeletal malformations. (**A**–**C**) CT scans of wild-type and *Fanci^−/−^* animals. *Fanci^−/−^* animals show cranial malformations (red arrows). (**D**) CT scans of *Fanci^−/−^* mice showing malocclusion. (**E**) Pictures and CT scans *Fanci^−/−^* animals showing limb defects, compared to wild-type littermates. Missing finger and fused remaining bones (top), and duplicated thumb (bottom) are shown.

We next evaluated wild-type, *Fanci^−/−^, Fancd2^−/−^* and *Fanci^−/−^Fancd2^−/−^* mice for hematopoietic phenotypes, including peripheral blood counts, bone marrow hematopoietic colony growth and hematopoietic stem and progenitor cell (HSPC) content. While whole blood counts and hemoglobin levels were similar in all animals (Figure 4D and F), peripheral blood platelet counts were lower in both single mutant mice and double mutant mice compared to the wild-type mice (Figure 4E). Interestingly, the bone marrow from double and single mutant mice contained comparable cellularities (Figure 4G) and progenitor cell numbers both by phenotype (Figure 4H) and by CFC assays (Figure 4H and I). Collectively, the double mutant *Fanci^−/−^Fancd2^−/−^* mice displayed similar hematopoietic defects as those observed in *Fanci^−/−^* or *Fancd2^−/−^* single mutant mice, suggesting an epistatic relationship between FANCI and FANCD2.

### Skeletal defects in *Fanci^−/−^* mice

More than 60% of FA patients are born with at least one physical abnormality, including thumb and arm malformation. Various possibilities have been reported: extra, misshaped or missing thumb and finger, but also incompletely developed or missing radius. To date, this phenotype has never been reported in any Fanconi mouse model. However, we observed that some *Fanci^−/−^* mice had cranial malformations compared to wild-type mice (Figure 5A–C). This malformation was associated with malocclusion, which occurs when the incisors overgrow because mandibular and maxillary teeth are not normally aligned (Figure 5D). Even more strikingly but observed with a low frequency (<5% of the total offsprings), *Fanci^−/−^* mouse displayed loss of one finger associated with bone fusion of other fingers, or duplicated thumb (Figure 5E). Such phenotype has not yet been previously reported in *Fancd2^−/−^*.

### Sterility and differential meiotic localization for FANCD2 and FANCI

**Figure 6.**
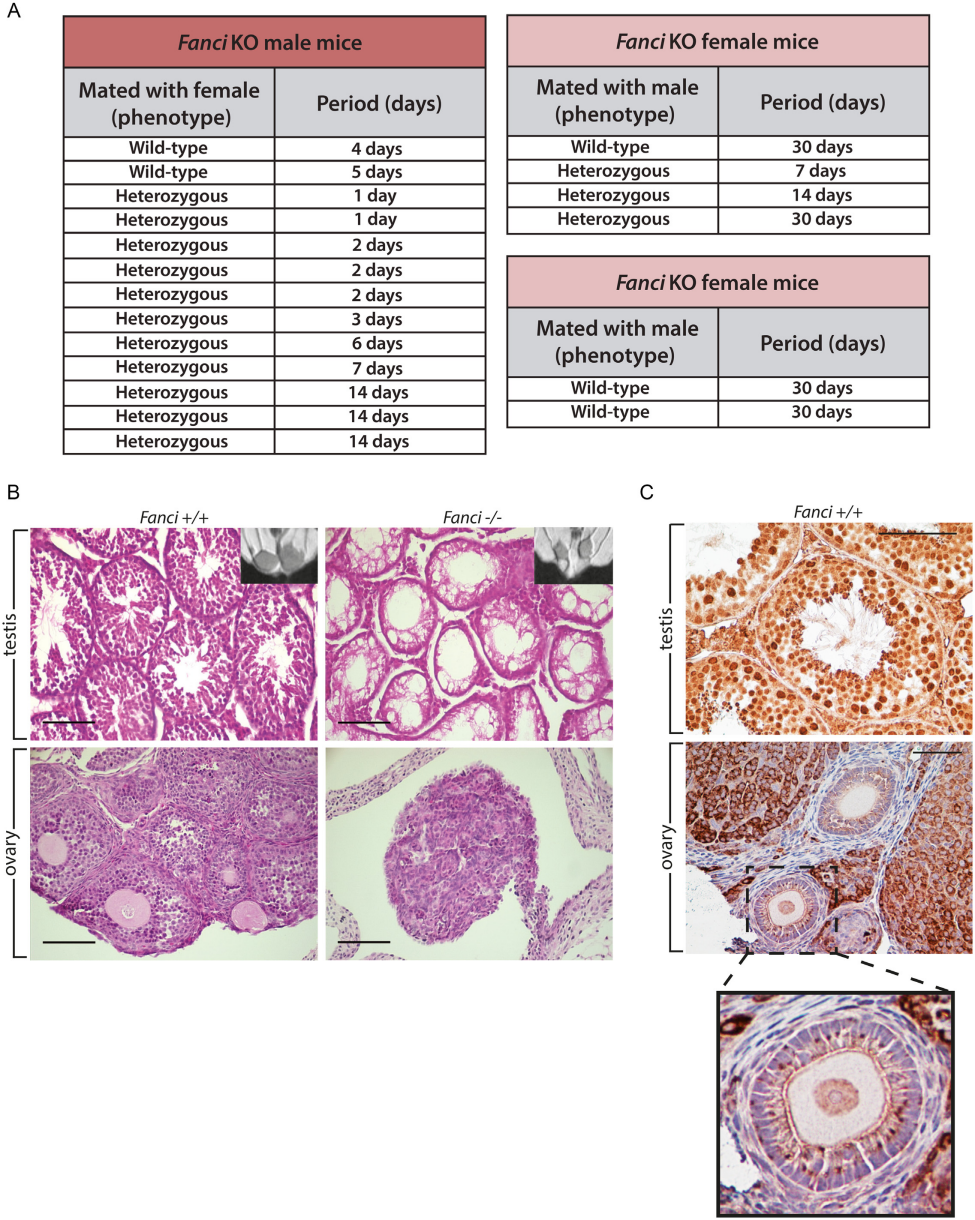
*Fanci^−/−^* mice are infertile. (**A**) Attempts to cross male and female *Fanci^−/−^* mice with wild-type or heterozygous *Fanci*^+/−^ mice do not lead to newborn mice. The period (days) relate to the time the indicated mice were in the same cage. (**B**) *Fanci^−/−^* mice show hypogonadism. Testis and ovaries from *Fanci^+/+^* and *Fanci^−/−^* animals were taken at 3 weeks, paraffin embedded and stained with hematoxylin eosin. All knockout animals were sterile because of a lack of germ cells. Scale bar corresponds to 100 μm. (**C**) Localisation of FANCI in gonads. Immunohistochemistry staining against FANCI on WT testis and ovaries.

We did not obtain any pups by breeding *Fanci^−/−^* mice together (Figure 6A and Supplementary Table S1), as opposed to *Fancd2^−/−^* animals (21). Magnetic resonance imaging (MRI) analysis revealed hypogonadism in (4-week-old) *Fanci^−/−^* males (Figure 6B). As ovaries are too small to be easily detected by this technique, we performed classical H&E histological staining on gonad sections, confirming a hypogonadism in all FANCI knockout animals (Figure 6B). *Fanci^−/−^* ovaries contained only medulla and stroma cells (Figure 6B). Likewise, male seminiferous tubules were only made of vacuolated Sertoli cells and no germ cells were detected, as judged by c-Kit staining which marks spermatogonial stem cells (41) (Supplementary Figure S5B–D). As a consequence, the failure of *Fanci^−/−^* males and females to generate mature gametes explained their sterility.

**Figure 7.**
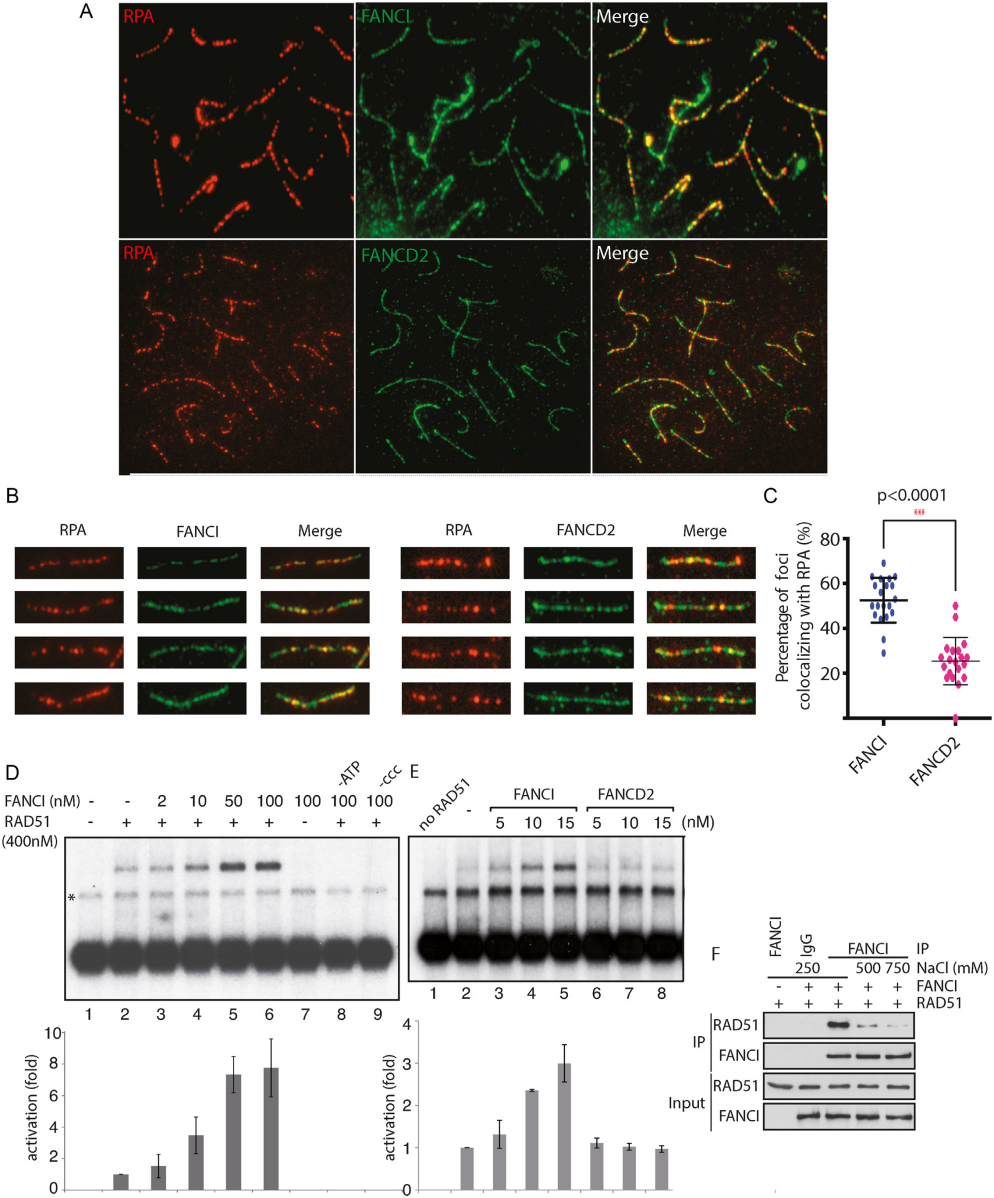
Discrepancies in FANCI and FANCD2 localization during meiosis. (**A**) Immunofluorescence was performed to detect RPA and FANCI or FANCD2 colocalization on meiotic spreads from WT mice. (**B**) A close-up view of representative pictures of the co-localization of RPA/FANCI or RPA/FANCD2. (**C**) Quantification of the percentage of colocalization between RPA/FANCI or RPA/FANCD2 on zygotene chromosomes (*n* = 30) (**D**) FANCI stimulates RAD51-mediated D-loop formation. D-loop reactions mediated by combinations of RAD51 (lanes 2–6 and 8–9) and FANCI (lanes 3–9). Lane 1, without protein. Lane 7, FANCI without RAD51. Lanes 8–9, FANCI-RAD51 reactions without ATP or supercoiled DNA (ccc), respectively. Bottom: quantification of the results in fold activation relative to RAD51 alone. The band indicated with an asterisk corresponds to annealed tailed molecules. (**E**) D-loop formation activity is specific to FANCI. D-loop reactions mediated by combinations of RAD51 (lanes 2–8) and FANCI (lanes 3–5), or FANCD2 (lanes 6–8). Lane 1, without protein. Bottom: quantification of the results in fold activation relative to RAD51 alone. (**F**) FANCI directly interacts with RAD51. Purified proteins were incubated together in buffer containing different salt concentration and immunoprecipitations were conducted using an antibody recognizing FANCI, followed by western blotting with anti-RAD51.

To confirm the localization of FANCI *in situ*, we performed immunohistochemistry staining on wild-type testes and ovaries. We found that FANCI was localized in spermatocytes and spermatids and in the nucleus of oocytes (Figure 6C). To gain further insights into the function of FANCI in spermatogenesis and meiotic recombination, we performed meiotic spreads. Since *Fanci^−/−^* mouse spermatocytes were not produced, it was not possible for us to distinguish whether FANCI affected the pairing of chromosome homologs. However, in wild-type tissues FANCI and FANCD2 localized all along the meiotic chromosomes in zygotene (Figure 7A). The localization of FANCI was similar to the one observed for RPA (42), being most abundant at early meiotic stages and decreasing thereafter at late pachytene (Figure 7A and B). In our hands, only rabbit polyclonal antibodies against FANCI or FANCD2 could be used, which precluded co-localization studies. FANCI and FANCD2 co-localized with RPA (Figure 7C), albeit at different levels, suggesting that both proteins participate in meiotic recombination.

### FANCI, unlike FANCD2, stimulates RAD51-mediated D-loop formation

To gain insights into a direct role for both FANCI and FANCD2 in meiotic recombination, we tested whether they were involved in the strand invasion step of HR. Stimulation of RAD51 D-loop formation by FANCI was tested with linear dsDNA (4.3 kb) with 3′tailed-ends (∼200 nt). Such substrates closely simulate long 3′-tailed DNA molecules *in vivo*, following the resection of DNA double-strand breaks (37). RAD51 was used at subsaturating concentration (400 nM), leading to weak D-loop formation (Figure 7D, lane 2). Interestingly, addition of FANCI to the RAD51 reaction resulted in 8-fold increase in D-loops (lane 6, 100 nM). The stimulation of RAD51 activity by FANCI was dependent on the presence of adenosine triphosphate (ATP) and supercoiled DNA (lane 8 and 9, respectively). FANCI did not promote strand invasion by itself (lane 7). In order to decipher if FANCD2 modifies the D-loop-promoting ability of FANCI, D-loop formation was also tested with FANCD2. In contrast to FANCI, at 15 nM, non-ubiquitinated FANCD2 did not promote RAD51-dependent D-loop formation (Figure 7E, lanes 2-4). Collectively, FANCI-specific stimulation of RAD51 in D-loop assays suggests that FANCI could interact directly with RAD51. Indeed, a complex between RAD51 and FANCI was observed by co-immunoprecipitation analysis (Figure 7F).

## DISCUSSION

One of the earliest cellular phenotypes of FA cells, hypersensitivity to oxidative stress, was described by Joenje's group 35 years ago (43). Since then, most studies on Fanconi anemia proteins have been conducted to clarify their role in the nuclear DNA damage response. As most of the phenotypes associated with Fanconi anemia cannot be studied in cell lines, we created a *Fanci* knockout mouse model to study its physiological roles.

In the present study, we report the first *Fanci* knockout model by creating a whole body disruption of *Fanci* exons 2 and 3 in the C57BL/6J mouse strain, leading to the absence of protein production. We observed similar phenotypes to existing FA mouse models: prenatal dwarfism, hypogonadism, sterility and sensitivity to DNA cross-link agents. Taking into account that FANCD2 and FANCI interact, we rationalized that if both proteins are only acting together via the ID2 complex, the *Fanci* knockout mouse should phenocopy the *Fancd2* knockout mouse. Indeed, this is the case for *Fanca^−/−^Fancc^−/−^* double-mutant mice, which are phenotypically identical to single mutants (44). Interestingly, our results suggest that the loss of FANCI induces both common and unique phenotypes when compared to FANCD2 (21,30), in the same C57BL/6J mouse background.

We demonstrate that *Fanci^−/−^* mice are partially viable, indicating that FANCI is not completely required for mammalian development. *Fanci^−/−^* and *Fancd2^−/−^* are not epistatic with respect to generating viable mice in crosses of double heterozygotes. Indeed, the embryonic lethality seems stronger in *Fanci^−/−^* mice as only 4.1% of mice reached weaning, compared to 16,5% for *Fancd2^−/−^* (21), 19.6 or 16.4% for *Fancc^−/−^* depending on the mouse strain used (45), 11% for *Slx4^−/−^* (32) or 6% for *Usp1^−/−^* (46). This is also in contrast to *Fanca, Fancg* and *Fancf* Mendelian ratios that are normal (19). Together with prenatal dwarfism and the diverse skeletal abnormalities we observed, one could conclude that FANCI is involved in embryogenesis, but not in an indispensable manner. Clearly, some phenotypes associated with FA, such as thumb duplication, might not be related to the DNA damage response. As shown in our study, one of the most prevalent phenotype observed in the *Fanci^−/−^* mouse was severe developmental aberration of eyes, also observed in *Fancd2^−/−^* mice (21). A recent study shows that the microphthalmia transcription factor (MiTF) is involved in *Fanca, Fancd2, Fancc* and *Fancg* gene expression (47). Waardenburg and Tietz syndromes are associated with mutations in MiTF, leading to partially similar phenotypes to FA, like deafness, missing bones, weak skin pigmentation and microphtalmia. This leads to the intriguing hypothesis that MiTF could also regulate *Fanci* to suppress microphtalmia.

Progressive bone marrow failure can be lethal in human Fanconi Anemia patients. FA mouse models are known not to develop anemia. However, many of the FA mouse models exhibit HSPC defects. *Fanci^−/−^* mice follow in the footsteps of *Fancc, Fancg, Fancd1, Fancd2* and *Usp1* mutant mice with normal peripheral blood counts (19,21,39,46,48,49). However, *Fanci^−/−^* mice have a smaller progenitor pool, and the bone marrow resembled *Usp1^−/−^* mice (46). Similar to *Fancd2^−/−^* mice (30), *Fanci^−/−^* mice also exhibit severe defects in HSPC function. It is interesting to note that the Lin-CD48+CD140-cells are in majority quiescent, and depleting FANCI has a lesser effect in this population suggesting that the effect is more important in proliferative HSC cells. Interestingly, FANCD2 and FANCI are epistatic for hematopoietic functions, suggesting that they may function in common molecular or cellular pathways required for maintaining hematopoiesis and may have identical functions in the bone marrow. Thus, our work highlights the critical role of FANCI in hematopoietic stem-cell survival and function.

Previous studies have shown that both FANCA and FANCC are expressed during limb embryonic development (50,51), but surprisingly none of the corresponding animal model, nor the others, developed limb abnormalities (19,51). In contrast, radial and thumb abnormalities are part of the most prominent FA phenotype (52). One of the intriguing phenotypes of our *Fanci*^−/−^ mice was the development of limb defects in some animals. To our knowledge, this is the first report of limb defects in a FA mouse model. As we observed limb defects in only a few animals (<5% of the total offsprings), the activation/inactivation of a pathway or modifier gene may be required. Interestingly, *p53^−/−^* and *p63^−/−^* mice showed limb defects, although with low frequency (53,54). *Fanci^−/−^* mouse limb defects may be linked to a p53-family-dependent pathway, otherwise modifier gene(s) might be involved. Modifier genes include spontaneous or wild-derived variations, or induced mutations captured in inbred mouse strains. The *Fancd2* mutant mice has an increased incidence of tumors of epithelial cell origin (21). At this time, although we have observed mixed types of neoplastic lesions in our *Fanci* mouse model, a large number of animals is required to conclude on these observations.

Although it was recently revealed that the FA pathway core complex proteins FANCA, FANCB, and FANCC are essential for FANCD2 foci formation on sex chromosomes (55), little is known on the function of FA proteins during meiosis. Several FA mutant mice exhibit a reduced number of germ cells in newborn mice and this was also observed for *Fanci^−/−^* mice. The impact of the depletion of FANCI on meiosis was significantly stronger than what observed in the *Fancd2^−/−^* mouse developed by Houghtaling *et al.* (21), but was similar to the double mutant *Usp1^−/−^Fancd2^−/−^* mouse (46). *Fanci^−/−^* mice were completely sterile, whereas *Fancd2^−/−^* crosses produced some animals (21). *Fanci^−/−^* mice produced no ovarian follicles, and spermatogenesis was completely impaired. The fertility phenotype was strongly penetrant alike *Usp1^−/−^* and *Fanca*^−/−^ mice, leading to a Sertoli cell-only (SCO) phenotype. Thus, our data suggest that FANCI participates in meiotic recombination in germ-cells. In support of this, we observed that FANCI directly interacts with RAD51 and stimulates RAD51-mediated D-loop formation. This is consistent with a recent report showing that FANCI-FANCD2 stabilizes the RAD51 nucleoprotein filament (56).

Throughout this manuscript we compared the phenotypes of the *Fanci^−/−^* mouse to the *Fancd2^−/−^* model generated by Houghtaling *et al.* (21). During the review process of this manuscript, the generation of a *Fancd2* depletion mice by CRISPR-Cas9 was reported (57). In this new model generated by Yang *et al.* (57), the deletion of exon 5 of *Fancd2* results in more severe phenotypes than the model generated by Houghtaling *et al.* which had deletion of exons 26–27 of *Fancd2* (21). Yang *et al.* report higher rates of embryonic and postnatal lethality, higher incidence of microphthalmia and more severe hypogonadism and only aberrant tubules with total absence of germ cells (57). Thus, we can conclude that the *Fanci* knockout mice have a more severe phenotype than the *Fancd2* knockout mice generated by Houghtaling *et al.* (21) but similar phenotypes as the Yang *et al. Fancd2*–/– mice model (57). Both Houghtaling *et al.* and Yang *et al.* generated *Fancd2*^–/–^ mice in the C57BL/6J background. Houghtaling *et al.* generated a targeted disruption of murine *Fancd2*^–/–^ with the deletion of exons 26-27. Using an antibody recognizing the N-terminus of FANCD2, they failed to detect a band of 88 kDa from testes, bone marrow or MEFs, corresponding to the predicted size of truncated FANCD2 (21). We therefore conclude from their data that the Houghtaling *Fancd2* model is also a complete knockout, and perhaps not a hypomorph. Thus, further comparative studies will be necessary to clarify these discrepancies.

In summary, we show that *Fanci^−/−^* mice exhibit many of the FA phenotypes including cellular hypersensitivity to DNA cross linking agents, fertility defects, skeletal defects and hematopoietic defects. Natural FANCI-deficient cases have been reported (58). Some cows develop the brachyspina syndrome, which is very similar to human FA. First, these cases are in accordance with some of our observations in the mouse model: death before birth, *in utero* growth delay, sterility and limb defects. This suggests that FANCI function is also conserved in cows, and corroborated by the high conservation of the protein sequence between mammals (12,20). Clearly, the study of FANCI functions is relevant for the diagnosis of cattle to diminish infertility or stillbirth cases. Furthermore, our studies also open the door to new therapeutic avenues for FANCI deficient patients. For instance, the diabetes drug metformin, was found to improve hematopoiesis and to delay tumor formation in *Fancd2*^−/−^ mice (59). Metformin might allow aldehyde detoxification (59). Hence, reducing the exposure to aldehydes might be also a therapeutic approach for FANCI-mutated patients.

## Supplementary Material

gkz514_Supplemental_Files
