# Supplementary material for: A Fanci knockout mouse model reveals common and distinct functions for FANCI and FANCD2
Source: Nucleic Acids Res. 2019 Jun 20;47(14):7532–47. doi: 10.1093/nar/gkz514 (PMC6698648; doi:10.1093/nar/gkz514)
Supplement: gkz514_Supplemental_Files [file gkz514_supplemental_files.zip › Dubois et al. Suppl Figures 2018.pdf]

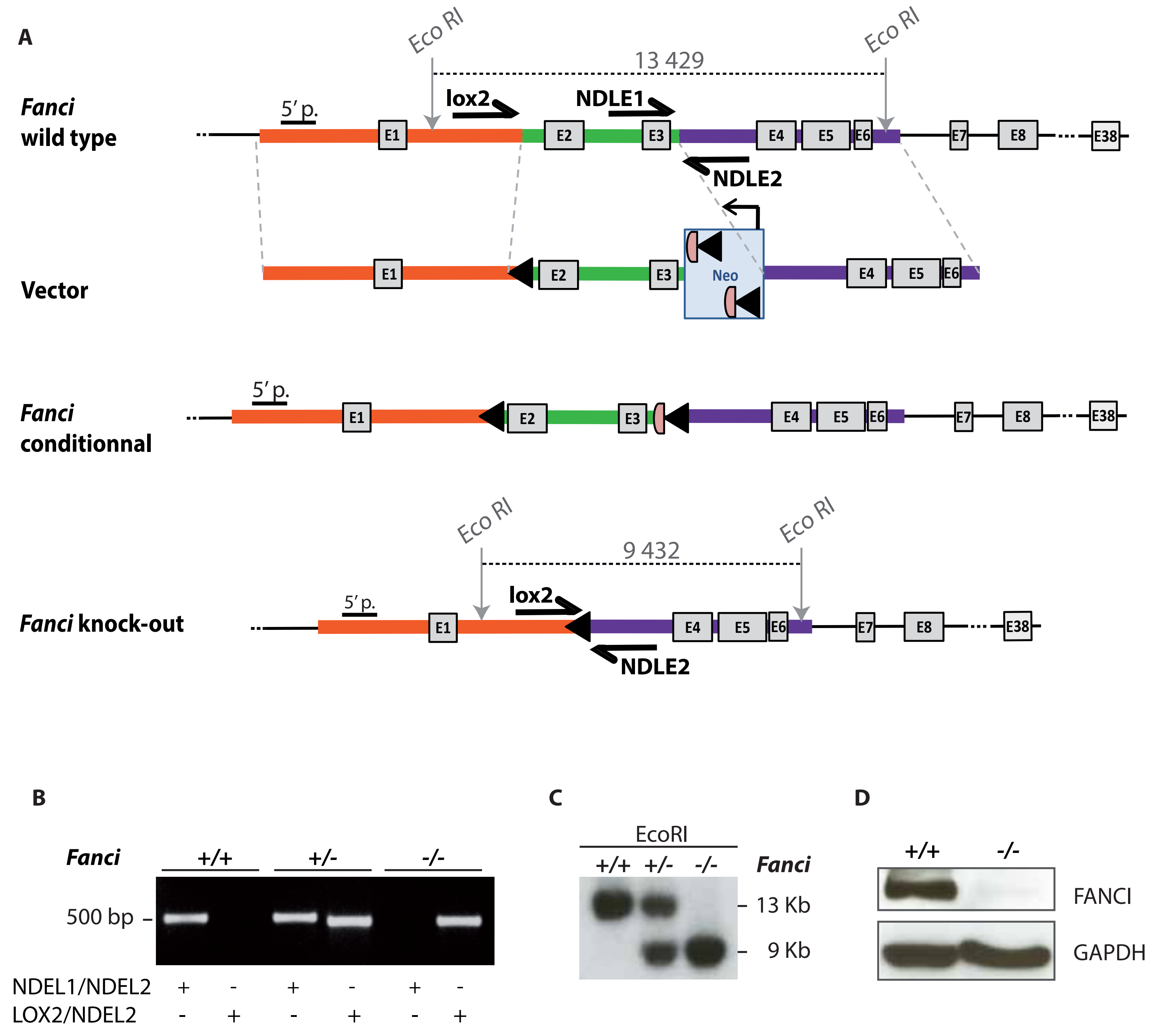
A
Eco RI
Eco RI
13 429
Fanci wild type
5′ p.
lox2
NDLE1
E1
E2
E3
E4
E5
E6
E7
E8
E38
NDLE2
Vector
Neo
Fanci conditionnal
Eco RI
Eco RI
9 432
Fanci knock-out
B
Fanci
+/+
+/-
-/-
500 bp
NDEL1/NDEL2
LOX2/NDEL2
C
EcoRI
13 Kb
9 Kb
D
FANCI
GAPDH

**Dubois E. *et al.*, 2018** **Supplementary Figure 2**

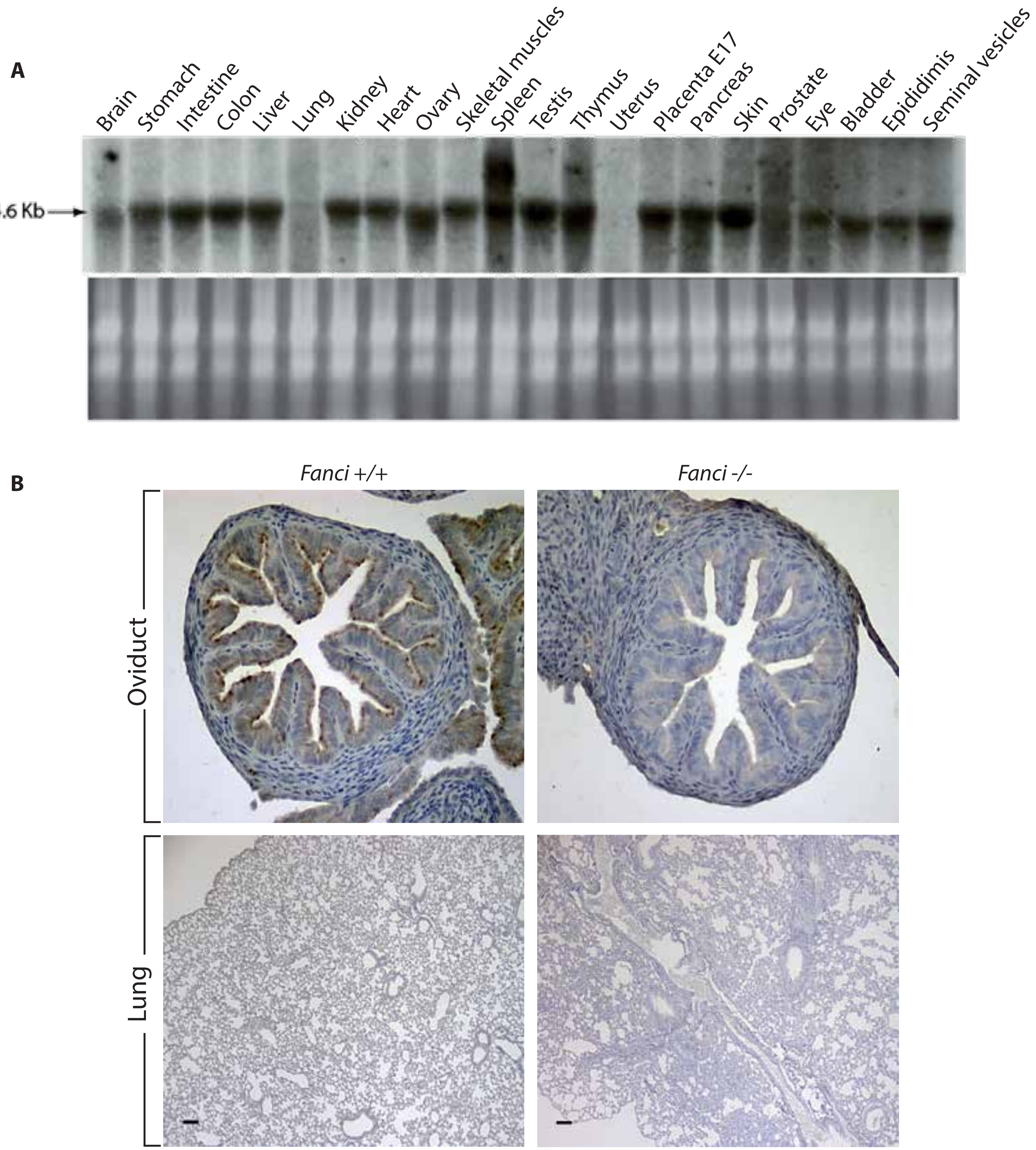

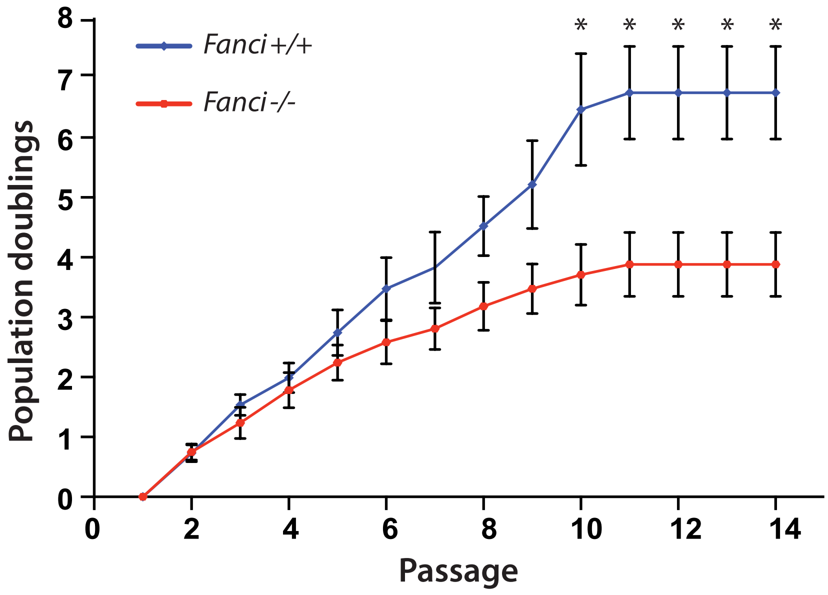

Fanci+/+
Fanci-/-
Population doublings
Passage
0
1
2
3
4
5
6
7
8
0
2
4
6
8
10
12
14
*
*
*
*
*

**Dubois E. *et al.*, 2018** **Supplementary Figure 4**

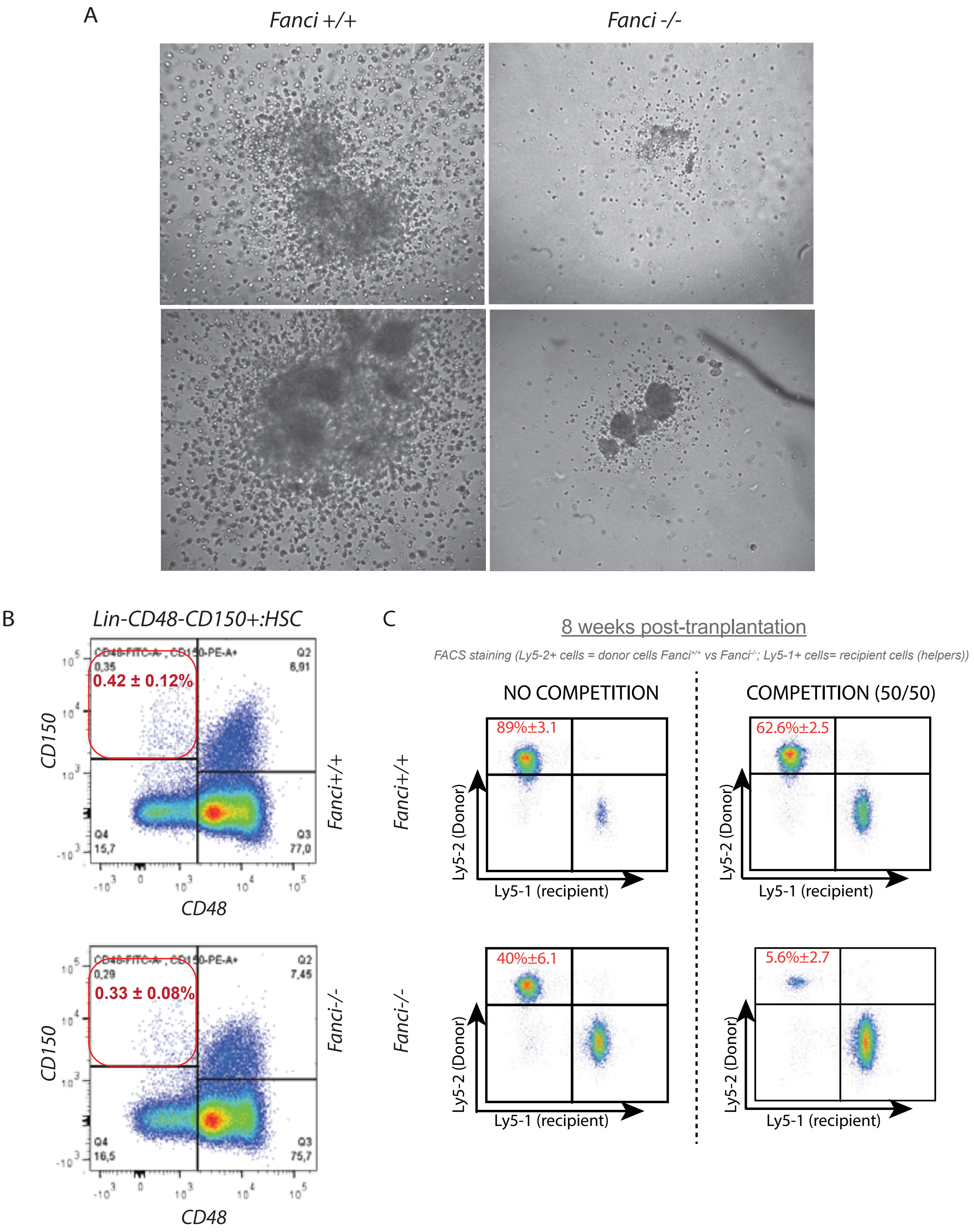

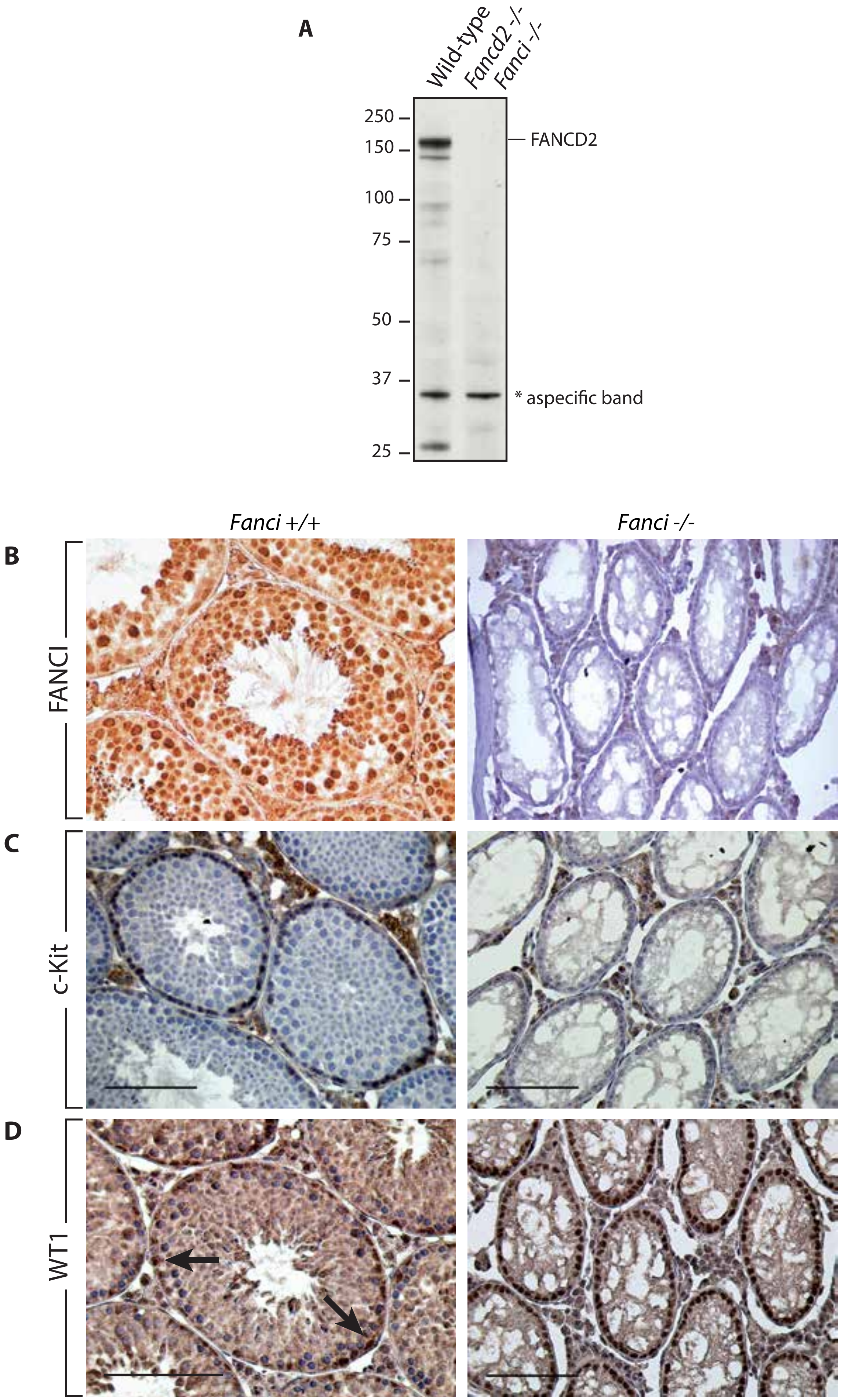
A
Wild-type
Fancd2 -/-
Fanci -/-
250
150
100
75
50
37
25
FANCD2
* aspecific band
Fanci +/+
Fanci -/-
B
FANCI
C
c-Kit
D
WT1

| ***Fanci* status** | ***Fancd2* status** | **# Offspring observed** | **% of the total number of offsprings** | **Mendelian % predicted** | **Significant difference between observed and predicted?** |
|---|---|---|---|---|---|
| +/+ | +/+ | 20 | 9.66 | 6.25 | Yes (p=0.0426) |
| +/+ | +/- | 28 | 13.5 | 12.5 | No (p= 0.6552) |
| +/- | +/+ | 35 | 16.9 | 12.5 | No (p= 0.0551) |
| +/- | +/- | 71 | 34.2 | 25 | Yes (p=0.0020) |
| +/+ | -/- | 16 | 7.7 | 6.25 | No (p= 0.3790) |
| -/- | +/+ | 7 | 3.4 | 6.25 | No (p= 0.0883) |
| +/- | -/- | 20 | 9.67 | 12.5 | No (p= 0.2169) |
| -/- | +/- | 8 | 3.86 | 12.5 | Yes (p= 0.0002) |
| -/- | -/- | 2 | 0.97 | 6.25 | Yes (p= 0.0017) |

*Table 1. Frequency of genotypes of offspring from interbred Fanci$^{+/-}$Fancd2$^{+/-}$ mice compared with predicted frequencies of expected Mendelian genetics.*
