# Supplementary material for: A Fanci knockout mouse model reveals common and distinct functions for FANCI and FANCD2
Source: Nucleic Acids Res. 2019 Jun 20;47(14):7532–47. doi: 10.1093/nar/gkz514 (PMC6698648; doi:10.1093/nar/gkz514)
Supplement: gkz514_Supplemental_Files [file gkz514_supplemental_files.zip › FANCI_Supplemental_information_NAR_April_2019-v2.pdf]

A *Fanci* knockout mouse model reveals common and distinct functions for FANCI and FANCD2

Emilie L. Dubois, Mariline Béliveau, Laure Guitton-Sert, Kalindi Parmar, Jalila Chagraoui, Julien Vignard, Joris Pauty, Marie-Christine Caron, Yan Coulombe, Rémi Buisson, Karine Jacquet, Clémence Gamblin, Yuandi Gao, Patrick Laprise, Michel Lebel, Guy Sauvageau, Alan d'Andrea, and Jean-Yves Masson

---

Supplemental Figure 1. Targeted disruption of the murine *Fanci* locus
(A) Map of the first 8 exons (represented by boxes) of the murine *Fanci* locus. The construct consists of 2 homology arms (orange and purple) flancking the targeted region (green). The promotorless Neo resistance cassette, inserted dowstream of exon 3 in the opposite direction relative to the gene, is surrounded by FRT sites (pink oval). In a *Fanci* conditional mouse, exons 2 and 3 can be removed by the Cre recombinase recognizing LoxP sites (black triangles). Primers for genotyping are indicated by black arrows (NDEL1, NDEL2, Lox2) and probe for southern blot by a black stick (5' p.). **(B)** PCR genotyping of mouse tails. +/+ wild-type ; +/- heterozygote ; -/- homozygous knockout. **(C)** The deletion of exons 2–3, about 4Kb, is confirmed by southern blot by using the 5' probe on genomic DNA extracted from mouse tails, digested with EcoRI restriction enzyme. The wild-type allele appears at 13Kb, although the knockout (KO) allele is about 9Kb. **(D)** Western blot with an anti-FANCI antibody (band around 150kDa) on wildtype and *Fanci* -/- testis protein lysate.

Supplemental Figure 2. (A) A premade membrane for Northern Blot from ZYAGEN company (« Mouse Multiple tissue Panel NB », Cat MN-MT-2) was used to analyze *Fanci* expression in 22 mouse tissues. The lower panel show total RNA in each lane. **(B)** Immunohistochemistry of FANCI on oviduct or lung tissues of wild-type and homozygous knockout *Fanci*-/-mice.

Supplemental Figure 3. (A) Heterozygote and homozygous knockout Mouse Embryonic Fibroblasts grow slower than wild-type MEFs. Mann and Whitney statistical analysis was performed (* $p<0.05$).

Supplementary Figure 4. (A) FACS analysis of Lin-CD48+CD140-cells in *Fanci*-/- mice compared to wild-type. **(B)** Colony Forming Cell assays in methylcellulose of wild-type and *Fanci*-/- hematopoietic progenitors. **(C)** FACS analysis of the competition and non-competition transplantation assays (the experimental scheme is provided in Fig. 3C, and data is related to figures 3D and 3E).

Supplementary Figure 5. (A) Western blot analysis of FANCD2 in *Fanci*-/- *Fancd2*-/- knockout mice. **(B-D)** Analysis of wild-type and *Fanci*-/- seminiferous tubules by FANCI, c-Kit (a spermatogonial stem cell marker), and WT1 staining (a sertoli cell marker).

Supplementary Table 1. Frequency of genotypes of offpring from interbred *Fanci Fancd2* mice compared with predicted frequencies of expected Mendelian genetics.
